# Community Awareness-Raising to Support Tobacco Policy: Results from an App-Based Photo-Mapping Activity with College Students

**DOI:** 10.1007/s10900-025-01490-6

**Published:** 2025-06-09

**Authors:** Megan E. Roberts, Brittany Alexander, Jade Davis

**Affiliations:** College of Public Health, The Ohio State University, 1841 Neil Ave., Columbus, OH 43210 USA

**Keywords:** Tobacco Retailers, Photo-mapping, Community awareness-raising, Public Support

## Abstract

**Background:**

The tobacco retailer environment (TRE) harms communities, but the implementation of policies to address it have been slow and uneven and receive tepid public support.

**Objective:**

To test whether a smartphone app’s photo-mapping activity could raise community awareness of the TRE.

**Methods:**

This was a non-randomized study with a pre-post design. For three weeks, 75 university students engaged with the app, which incentivized participants to submit photos of significant aspects of tobacco in their environments (e.g., tobacco litter, tobacco retailers). Surveys administered before and after the app period assessed tobacco- and policy-related perceptions.

**Results:**

Repeated-measures analyses indicated that, over the course of the study, participants increased in their awareness of tobacco advertising, awareness of tobacco litter, anti-tobacco industry attitudes, and support for an outdoor tobacco-free campus policy. Those most engaged in the study (as indicated by the total number of photo submissions) also increased in their attitudes about there being too many tobacco retailers.

**Conclusions:**

Overall, findings suggest an app-based, photo-mapping activity could be an innovative means of directing people’s attention to the impact of the tobacco industry in their local community. Such activities could help increase public support for tobacco retailer licensing and other TRE-focused policies.

## Background

 The issue of the tobacco retailer environment (TRE) is a well-documented concern across the globe. In the U.S., there are over 1.22 tobacco retailers (i.e., any type of store that sells tobacco, including convenience stores and tobacco shops) per every 1,000 persons [1]. Tobacco retailers provide access to tobacco products, expose passers-by to tobacco marketing, and accumulate tobacco litter [2–4]. Numerous studies demonstrate that youth and adults living in areas with a higher density of tobacco retailers have a higher likelihood of tobacco use [5–10] and a lower likelihood of cessation success [11–15]. Thus, the tobacco retailer environment plays a major role in perpetuating a society’s state of tobacco use and nicotine addiction.

In recognition of the public health impact of the TRE, policies have been introduced to reduce the density of tobacco retailers, such as tobacco retailer licensing (TRLs). For example, many U.S. cities, including San Francisco, Philadelphia, and New York City, have imposed restrictions such as prohibiting the sale of tobacco retailers close to schools, restricting how close tobacco retailers can be to one another, or capping the total number of retailers allowed in an area. Preliminary evidence indicates these policies are effective at reducing density [16, 17] and have the potential to be equity-enhancing [18], meaning they would reduce density in the historically marginalized communities where density is often highest. However, progression has been slow and uneven. In Ohio, for example, only 13.7% of census tracts have implemented TRLs, and nearly all were in urban or suburban areas [19]. Even California, one of the most progressive states in terms of tobacco control, has zero TRLs within 23 of its 58 counties [20].

To continue the adoption of policies such as TRLs, public awareness and support for a policy can be critical. Strong public support may increase priority among policymakers, especially when the public is mobilized and the support is impassioned [21]. Thus, generating public support and mobilizing citizens on an issue can be a powerful advocacy tool [22, 23]. Raising public awareness and support is also a matter of ethics: community members should be included in the conversations surrounding their community’s health-related issues and the proposed solutions.

The purpose of this project was to assess an interactive, “gamified” means of raising community awareness of the TRE. Specifically, we used an app that allowed participants to both submit photos of significant aspects of tobacco in their environments (e.g., tobacco litter) and view the mapped record that they and all participants were building of submitted photos. We envisioned that engaging in this app-based, photo-mapping activity would provide a novel means of directing participants’ attention to the impact of the TRE in their local community. Similar types of photo-mapping and photovoice activities have been used in other community engagement work [e.g., 24–27]; however, reports on these activities are generally qualitative in nature. For the present study, we conducted pre-post analyses to quantify how much our activity impacted participants’ TRE-related awareness, attitudes, and policy support.

## Methods

### Participants

We recruited a convenience sample of 113 participants, aged 18 or older, at our university. Of these, 26 did not continue with the study after baseline, leaving 87 participants for analysis. Our University’s IRB approved all study procedures.

## Procedures

This was a non-randomized study with a pre-post design. Participants first completed an online consent form and a brief baseline survey about their tobacco and policy-related perceptions (see measures section below). Next, in a Zoom training session, a study staff member instructed participants on how to use the crowdsourcing app. Participants then began their 3-week crowdsourcing period, where they used a smartphone app (https://www.poketapp.com) to take photos of key aspects of tobacco in their day-to-day environments. Participants were instructed to make photo submissions as they went about their daily lives and to upload these photos to the app in real-time, allowing the photo to be tagged and mapped to its geolocation. Photo submissions were reviewed and “approved” by study staff on a rolling basis, to confirm that they met submission criteria (incremental payments were used to encourage ≥ 20 approved submissions per week). The crowdsourcing feature of the app allowed participants to view on their maps the geolocated reports of other (de-identified) participants. Upon completion of their 3-week data collection period, participants completed an online, follow-up survey that re-assessed tobacco and policy-related perceptions and evaluated their study experience.

## Survey Measures

Participants first reported about their use of various tobacco products. Responses were aggregated to create a measure of ever use of any tobacco product (yes/no) and past-30-day use of any tobacco product (yes/no).

A single-item measure asked, “In your daily life, how much do you notice advertisements for tobacco products?” (from 1 = *not at all* to 7 = *very much*). Similar questions asked about noticing advertisements for alcohol products and sugary beverages like Coke or Gatorade.

Using items adapted from the Legacy Media Tracking Survey [28], we assessed *anti-industry attitudes* with statements about tobacco industry deception and harm and *advocacy-based attitudes* with statements about wanting to see an end of the tobacco industry. Two additional attitude statements were: “Too many stores sell tobacco in Columbus” and “There is too much tobacco litter on and around campus.” We also assessed agreement with two tobacco policies that directly impact students’ communities: “The Ohio State University campus should be tobacco free in all buildings” and “The Ohio State University campus should be tobacco free including all outdoor areas” (for all items: 1 = s*trongly disagree* to 5 = *strongly agree*).

At the end of the baseline survey, we assessed age, gender, race, and ethnicity. The follow-up survey re-assessed the items described above concerning advertisement noticing, attitudes, and policy support. We also asked questions to evaluate the study experience.

## Crowdsourcing App Measures

The crowdsourcing app allowed participants to complete a survey at any time and as many times as they wanted. Available submission types were *Tobacco Retailer*,* Tobacco Litter*, or *Community Location.* With each survey submission type, participants uploaded a photo: the tobacco retailer, the tobacco litter, or the community location.

## Results

The mean age of our sample was 21.1 (SD = 2.41); 73.6% were female and 60.9% were non-Hispanic White. Consistent with our quota sampling, 48.3% had never used tobacco, 27.6% had a history of tobacco use but were not currently using, and 24.1% currently used tobacco. Among those currently using a tobacco product, e-cigarettes were the most prevalent, but participants also reported use of cigarettes, cigars, and nicotine pouches.

Twelve participants (13.8%) dropped out of the study during the crowdsourcing period, leaving a sample of 75 for pre-post analyses (attrition was higher among males, but was not related to any other sociodemographic characteristics or tobacco use history). Among the 75 participants who completed all components of the study, the number of submissions that a participant provided over the 3-week crowdsourcing period varied greatly, ranging from 12 to 106 (mean = 56.6, SD = 19.94, median = 60). The average prevalence of litter submission among participants was 80.4% (community locations was at 10.5% and retailers at 9.1%).

**Fig. 1:**
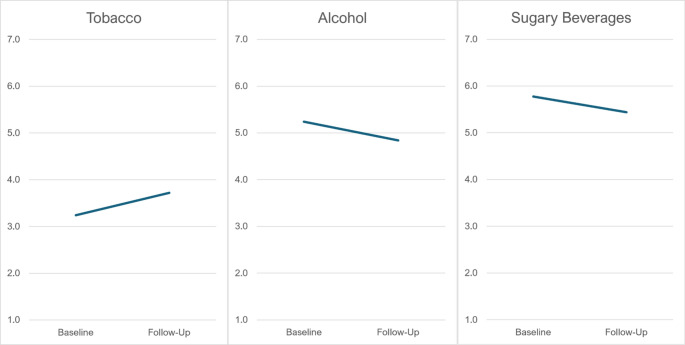
Participant reports of the extent to which they notice advertisements for tobacco, alcohol, and sugary beverages, reported at baseline and follow-up. Higher values indicate more noticing

Repeated-measures ANOVA comparing pre-post survey responses indicated that noticing of tobacco advertisements increased over the study, F(1, 74) = 10.4, *p* =.002 (Fig. 1). We observed the opposite trend for our control questions: noticing alcohol advertisements declined over the study (*p* =.013), and there was no difference for noticing sugary beverage advertisements (*p* =.41).

Anti-industry attitudes increased over the study (F(1, 74) = 6.46, *p* =.013). However, there was no significant change in advocacy-based attitudes. There was a substantial increase in the attitude that there was too much litter (F(1, 74) = 30.9, *p* <.001). Change in the attitude that there were too many tobacco retailers was more complex: there was a significant Time×Submissions interaction, such that beliefs about too many tobacco retailers only increased for participants with a high number of submissions.

At baseline, support for an indoor tobacco-free campus policy was very high, with a mean of 4.6 (SD = 0.86) on a 1–5 scale. Baseline support for an outdoor tobacco-free campus policy was lower, with a mean of 3.9 (SD = 1.35). Pre-post analysis indicated no significant change in support for the indoor policy, but a significant increase in support for the outdoor policy (F(1, 74),=7.65, *p* =.007).

Evaluation data in the follow-up survey indicated that participants generally enjoyed being in the study (M = 4.1, SD = 0.71) and did not perceive it to be too much effort (M = 2.9, SD = 0.94). The majority (57.3%) reported that study participation took an hour or less of their time per week. The most common reason for difficulty with completing the weekly submissions was “Being too busy with other things” (22.7% reported “yes, a little” and 52% reported “yes” or “yes, a lot”). The least common reason was “Not finding things to report about” (26.7% reported “yes, a little” and 13.3% reported “yes” or “yes, a lot”).

## Discussion

To our knowledge, this is the first study to quantitatively evaluate how a photo-mapping activity changes participant-reported awareness, attitudes, and policy support. Overall, we found that participation in this app-based photo-mapping activity increased awareness of tobacco advertising, increased awareness of tobacco litter, increased anti-tobacco industry attitudes, and increased support for an outdoor tobacco-free campus policy. For those most engaged in the study (as indicated by the total number of submissions), participation also increased attitudes about there being too many tobacco retailers. Importantly, participants generally found this study to be enjoyable and not too time-consuming. This “gamified” method of community mapping, used with teens or adults, could provide a starting point for discussions about the tobacco environment in one’s community, as well as retailer-focused policy strategies.

Of note, although anti-industry attitudes increased for this study (i.e., viewing the tobacco industry as harmful and deceptive), we did not observe an increase in advocacy attitudes (i.e., a desire for active involvement in getting rid of tobacco). One potential reason for the lack of change in advocacy attitudes is that our method did not provide any information about how these actions could be undertaken. In other words, although the crowdsourcing procedures directed participants’ attention toward industry harms like tobacco litter and tobacco retailers, it did not present steps that an individual could take to counter those harms. Future methods could consider including information or tools for taking such steps, thereby empowering individuals to take community action.

This study also observed no change in support for an indoor tobacco-free campus policy. We believe that this null effect was simply due to a ceiling effect. Specifically, whereas support for an outdoor tobacco-free campus policy was moderate at baseline (3.9 on a 5-point scale) and had room to significantly increase at follow-up, support for an indoor tobacco-free campus policy was already quite high (4.6 on a 5-point scale) and had little room to significantly increase.

Limitations to this study include attrition and our lack of a control condition. One unexpected outcome of this study was that the number of tobacco retailer submissions was low (9%). This was possibly because students spent much of their time on a central campus, where there are no tobacco retailers. Future versions of this study should be conducted with more heterogeneous communities and more heterogeneous neighborhoods. Future community-based approaches should also consider facilitating a group reflection session following their crowdsourcing period, where participants can discuss their experiences, review their crowdsourced maps, and further reflect on the extent of the tobacco industry’s presence in their community.

One unexpected outcome of this study was that the number of tobacco retailer submissions was low: the average prevalence of tobacco retailer submissions among participants was 9%, whereas the average prevalence of tobacco litter submissions among participants was 80%. The focus on litter was possibly a product of students spending much of their time on a central campus, where there are no tobacco retailers. Thus, finding litter examples may have just been easier. Future studies or programs utilizing this app-based photo submission method could consider quotas, whereby participants are required to provide a certain number of each submission type. Additional submission types could also be included, such as tobacco advertisements.

## Conclusions

Retailer-focused policies, such as TRLs, can be an effective means of reducing tobacco retailer density—thereby advancing health promotion and disease prevention. However, policy progress faces many barriers, including lack of political will and low public support. The present study suggests that an app-based, community-mapping approach may be an effective means of engaging communities, drawing their attention to the impact of the tobacco industry in their environment, and changing their tobacco- and policy-related perceptions. Overall, this approach could serve as a useful tool when laying the groundwork for policy advocacy.
